# Culture in salutogenesis: the scholarship of Aaron Antonovsky

**DOI:** 10.1177/1757975914528550

**Published:** 2014-12

**Authors:** Carina Benz, Torill Bull, Maurice Mittelmark, Lenneke Vaandrager

**Affiliations:** 1. Wageningen University – Health and Society, Wageningen, Netherlands; 2. University of Bergen – Department of Health Promotion and Development, Bergen, Norway

**Keywords:** salutogenesis, culture, health promotion, Antonovsky

## Abstract

Aaron Antonovsky wrote extensively, although disjointedly, about the roles of culture in salutogenesis. This paper provides a synopsis of his work in this arena. A literature review identified those of his English language writings in which culture was a subject, and relevant text segments were analysed using an inductive followed by a deductive method. Using thematic network analysis, text segments were sorted inductively by open coding and then analysed. This was followed by deductive text segment coding guided by the constructs of the salutogenic model of health. The analysis revealed that Antonovsky had an expansive interest in the roles of culture in salutogenesis. His writings included attention to the role of culture in: (a) shaping life situations; (b) giving rise to stressors and resources; (c) contributing to life experiences of predictability, load balance and meaningful roles; (d) facilitating the development of the sense of coherence and (e) shaping perceptions of health and well-being. Antonovsky’s writings about culture were sometimes conjectural, as well as being obviously influenced by his life experience in the USA and then in Israel, and by the spirit of the times in which he lived. However, he also drew extensively on his own and others’ empiricism, leading him to view culture as an integral aspect of the salutogenic model of health. The present analysis provides salutogenesis scholars with a roadmap of Antonovsky’s reflections, ponderings and conclusions about culture in the context of salutogenesis. It provides assistance in the form of an overview of Antonovsky’s treatment of culture in the context of salutogenesis.

## Introduction

This paper presents an analysis of how the concept ‘culture’ is treated in the writings of the medical sociologist and anthropologist Aaron Antonovsky, the originator of the famous salutogenic question, ‘what are the origins of health?’ His answer was the Salutogenic Model of Health (SMH), with the Sense of Coherence (SOC) at the core (1). He wrote extensively about how one’s life situation influences the development of the SOC, with broad-ranging attention to culture, social forces, social position, gender, ethnicity, age, genetics and plain luck, among other factors (2–5). Culture, in particular, is an omnipresent theme in Antonovsky’s writings, and he repeatedly signalled its importance to the development of the SOC. He also invited scholars to generate *other* answers than that of the SOC to the salutogenic question (6).

For those in search of other answers, as well as deeper understanding of the SOC, the study of culture seems to be of fundamental importance. One natural starting point is Antonovsky’s own wide-ranging observations and theorising about culture. Yet, while he addressed culture in all his books and most of his journal articles, the accumulated work is disjointed. Antonovsky never gathered his ideas about culture’s roles in salutogenesis in a dedicated manuscript, and we do not know if he had intentions to do so. Regardless, his death at age 71 abruptly ended a period of especially high productivity, and left it to posterity to assemble the pieces about culture into a whole, a task not undertaken until now, as far as we are aware.

Thus, the aim of this paper is to provide assistance to health promotion practitioners and researchers, in the form of an overview of Antonovsky’s treatment of culture in the context of his collected works on salutogenesis.

## The salutogenic model of health

**Figure 1.**
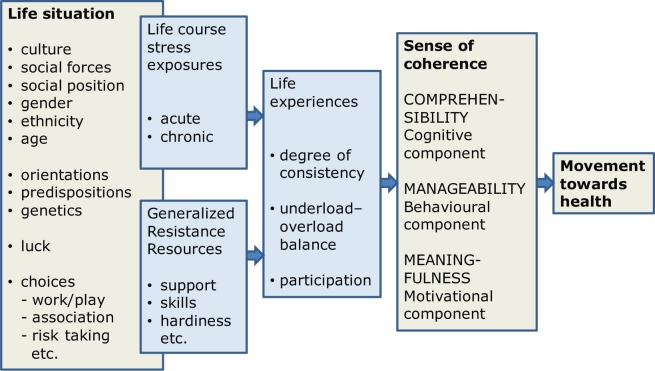
The salutogenic model (7), based on Antonovsky, 1996 (1).

The analysis presented in this paper is framed in large part by the SMH. Figure 1 shows the SMH in a highly simplified form (7). In this depiction of the SMH, and in Antonovsky’s much more detailed depiction in *Health, Stress and Coping* (3) and reproduced in Mittelmark and Bull (8), culture is a fundamental determinant of one’s life situation, and the arrows indicate that culture exerts influence on movement towards health through multiple paths and mechanisms. It is the task of the present analysis to develop a coherent and explicit statement about those paths and mechanisms, resulting in a roadmap that can be used to navigate Antonovsky’s scholarship on culture and salutogenesis.

## Methods

### Data collection

**Figure 2.**
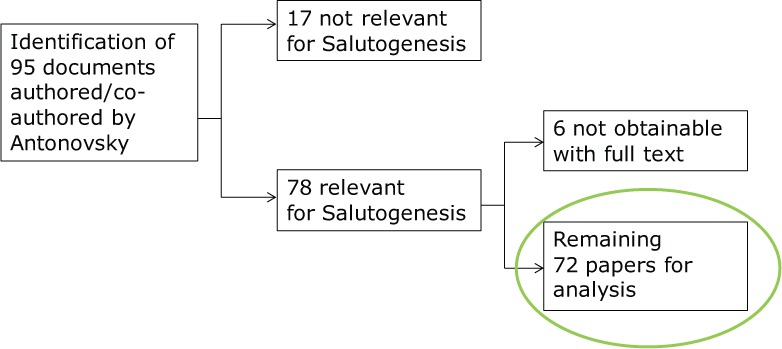
Flowchart data analysis.

The data used in this analysis are from all available papers published by Antonovsky in English, as first or co-author, in journals and in books. The databases searched using author search options were Web of Science, PUBMED, Cinahl, ERIC, EMBASE and PsychINFO. Works in Hebrew were excluded (even though the authors could have obtained collaboration to analyse the Hebrew papers) to enhance the transparency of the analysis and its interpretation, considering the international readership of this journal. A comment about inclusion criteria is in order. Antonovsky often made assertions having an empirical base (his own and others’ scholarship), but just as often his assertions were based on conjecture founded in his impressions and assumptions. We examined conjectural as well as empirical material, as our aim was not to restrict the analysis to what he *knew* about culture and salutogenesis, but rather to open the analysis to what he *thought and believed* as well as what he knew. In total, 95 documents authored or co-authored by Antonovsky were identified. Of these, 17 were not relevant to salutogenesis. Of the remaining 78 papers, six could not be obtained with full text, and 72 (92%) were obtained and analysed (see Figure 2). These 72 papers are collected in a database in the form of searchable PDF files.^i^

### Data analysis

The data analysis took place in two stages. Stage 1 was a Thematic Network Analysis (TNA) (9), which is a well-described and much applied inductive analysis, see for instance Attride-Stirling (10) and Skovdal *et al*. (11). All documents were searched for the words ‘culture’ and ‘cultural’. Text segments with these words were entered into the software NVivo for qualitative data management, and basic themes were composed using the open coding method. Clusters of basic themes were then sorted into organising themes, all relating to ‘culture’ as the global theme. In stage 2, the analysis moved to a deductive approach in which the basic themes from Stage 1 were sorted by the elements of the simplified SMH in Figure 1.

## Results

**Figure 3.**
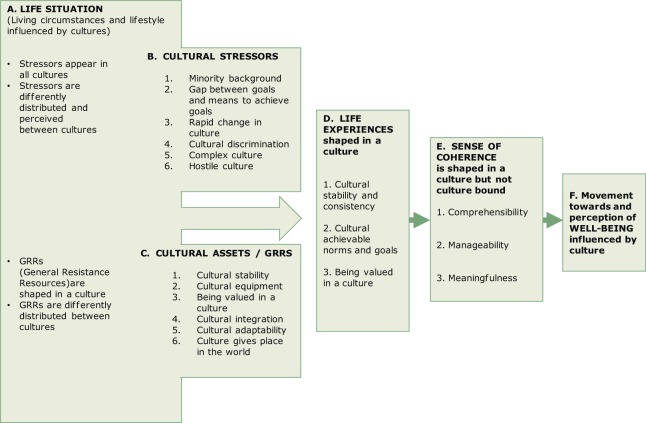
The roles of culture in the salutogenic model of health.

The TNA in Stage 1 revealed four organising themes. Antonovsky wrote about culture: (a) in his role as a medical school Professor; (b) as an immigrant to Israel; (c) as an anthropologist; and (d) as the originator of salutogenesis. This paper is restricted to a deeper analysis of the data in the salutogenesis organising theme. Therefore, the Stage 2 analysis focused on the fourth organising theme, above, sorted into the elements of the simplified SMH as shown in Figure 1. The sections below are sub-titled A–F in accordance with Figure 3.

### A. Life situation

Following Figure 3 from the upper left, Antonovsky wrote that while *stressors* are ubiquitous in all cultures, they are differently distributed between cultures, and seemingly identical stressors are perceived differently from culture to culture (2,3). Illustrating this, he was of the view that there are fewer stressors in southern compared to northern countries (2,4). From his multicultural climacterium research (12), he noted that the degree to which menopause is perceived as stressful varied as a function of culture. However, he wrote much more extensively about culture as a stressor in *itself*, than about cultural differences in exposure to and reaction to stressors.

### B. Cultural stressors

In some of his earliest research in the USA, long before he articulated the salutogenic question (what are the origins of health?), Antonovsky observed that youth from minority backgrounds had lower aspirations than youth living in the culture into which they were born, which he termed the ‘ethnic handicap’ (13). More than two decades later, on the subject of ethnic handicap, he wrote:

The more the integration, the greater the ease in making sense of the messages.^ii^ These are the core problems of minority groups, immigrant workers, lower class persons, illiterates […] and of women. To the degree that they cannot escape into a viable subculture, they are bombarded by noise and by brutal information. (14:99)

In the same vein, he noted that the problem for people who are not integrated is not only that they are constantly confronted with new information, but also that they do not have the legitimacy to be heard (5).

Addressing other manifestations of the ethnic gap phenomenon, Antonovsky wrote about how a gap between goals set by social expectations and one’s means to achieve those goals is an important cause of cultural stress (15). In *Health, Stress and Coping* (3) he addressed this issue briefly, positing that people from lower class America were stressed because they are taught the ‘American dream’ but are not able to live it (14). Exacerbating the potential for stress, he noted, was rapid culture *change*, which could have adverse health consequences due to the difficulty of adjusting to society’s new values, demands and expectations (3).

Another expression of cultural stress that received Antonovsky’s attention emanates from the *complexity* of culture, with cultures of high complexity, which signal many norms and rules, heightening the experience of stress (5). Aside from the untoward effects of cultural complexity, Antonovsky wrote about culture *hostility*, remarking that people who are confronted with arbitrariness and chance, and are controlled by hostile powerful others, are constantly under stress that is exacerbated by their powerlessness (14).

### C. Cultural assets/generalized resistance resources

Antonovsky addressed culture not only as a source of stress, but also as a key source of Generalized Resistance Resources (GRRs). A particularly important aspect of this, which Antonovsky commented on in many of his works, is cultural stability. The earliest instance of his attention to cultural stability was findings from his and colleagues’ investigation in Israel of adaption to menopause (16,17). Five ethnic groups participated: one Arab group and four Jewish groups with Central European, Persian, Turkish and North African origins. Findings from medical examinations revealed no differences among the groups in somatic symptoms, while the level of complaints about psychosomatic symptoms did differ: the Persian and North African groups had higher levels of complaints than the Central European and Arab groups. Antonovsky and his colleagues hypothesised that the crucial factor explaining these findings was the degree of cultural stability of the various ethnic groups and their degree of adaptation to their present situation in Israel (16).

Even if the study just mentioned predated Antonovsky’s first writings to address salutogenesis explicitly, it clearly was part of the foundation of his interest in cultural stability as a key GRR, as a factor in the building of a strong SOC, and contributing importantly to well-being:

Clearly, if one has a high intelligence, lots of money, or a clear ego identity or lives in a stable, integrated culture – to mention some GRRs – there will be consequences not only for the emergence of a strong SOC, and therefore health, but for other areas of well-being as well. (2:181)

Some degree of interpretation is needed to come to grips with Antonovsky’s ideas about culture’s roles in building GRR, since his use of terminology changed somewhat over the long period of his scholarship. An important instance of this is his early description of ‘cultural equipment’ (18). This is a concept that he probably would have referred to as a GRR when he began writing about salutogenesis. Writing about cultural equipment, he noted that adolescents need to grow up in a culture that supports them to develop their individual potential, that provides them with success models that they can look up to, and that provides them with tools (equipment) to reach their aspirations (18). We find no writings wherein Antonovsky deepened his ideas on this subject under the label ‘cultural equipment’, but the core of this concept came up repeatedly in his writings about GRR.

Quite interestingly, Antonovsky dwelled on the idea that one’s own role as a GRR in others’ lives is a GRR for *oneself*. He raised this idea in his discussion of Holocaust survivors in Israel who developed meaningful and respected social roles in Israeli society (15). This is one form of cultural *adaptability*; another is the ability to adapt to cultural norms, which is a source of strengthened SOC (3). Although Antonovsky described a complex culture as a potential stressor, he also wrote that a complex culture offers many possibilities of choice, and can thus be supportive for people who are flexible (5). Yet another form of cultural adaptability is successful cultural *integration*, which has salubrious effects that are the opposite of the deleterious effects of cultural discrimination (5). Summing up this section of results, Antonovsky made it quite clear that for him, culture plays a key role in the SMH:

[…]a culture provides its members, group and individual, with ready answers, clear, stable, integrated; with keening for a death, an explanation for pain, a ceremony for crop failure, and a form for disposition and accession of leaders. At the other extreme, which at times becomes a reality for individuals and groups, there is only utter chaos; there are no answers. Ready answers provided by one’s culture and its social structure are probably the most powerful GRR of all. (3:118–119)

### D. Life experiences and E. The sense of coherence

Moving to the right side of Figure 3, Antonovsky wrote that a supportive culture provides people with the life situation, the resources and thus with the life experiences needed to perceive life as comprehensible, manageable and meaningful, the three elements integral to the SOC.*Comprehensibility* arises from a stable culture that sends consistent messages to people, *manageability* is created by a culture that gives people the tools to live up to norms set by the culture, and *meaningfulness* is supported by a culture that values the role of people and gives them a place in the world (2). In addressing the connections between culture and the SOC, Antonovsky repeatedly expressed his hope and belief that the SOC is a cross-culturally meaningful construct (1,2,19,20). He wrote:

The SOC is hopefully, a construct […] which is universally meaningful, one which cuts across lines of gender, social class, region and culture. It does not refer to a specific type of coping strategy, but to factors which in all cultures, always are the basis for successful coping with stressors. (20:726)

In concert with this position, Antonovsky often returned to his thesis that there are many cultural roads to a strong SOC (3,21,22). However, he also cautioned that not all cultures provide the same conditions for a strong SOC: ‘Once again it must be stressed: there are many cultural paths to a strong SOC. This does not mean that all cultures and subcultures are equally conducive to a strong SOC’ (2:94).

### F. Movement towards and perception of well-being influenced by culture

Finally, moving to the far right of Figure 3, Antonovsky wrote that apart from culture’s role in influencing health via the paths described above, culture also influences one’s notions about what it means to be healthy,^iii^ as he observed in his research on the climacterium among women (17,23).

## Discussion

Antonovsky grew up in New York as the son of Russian-Jewish immigrant parents, and later immigrated to Israel. He often described Jewish culture as being stable and supportive, fulfilling all the requirements for reaching a strong SOC (e.g. Maoz *et al*., 1977 (17)). In contrast, he viewed the USA as a country wherein people are confronted with goals set by the media, which most of them cannot achieve (15). He was quite transparent in his use of these lenses in his analysis of stress and coping. What Antonovsky *knew* about culture and what he *felt* about culture were inextricably intertwined in his writings taken as a whole.

Antonovsky also wrote as *a man of his times and his training*. He was a stress researcher in the 1950s and 1960s, focusing on heart disease, an important malady in the discourse revolving around the stress of modern living. It is on this backdrop, it seems, that he maintained that stress exposure is lower in southern compared to northern countries, a belief that has later been contradicted by empirical research (e.g. Eriksen *et al*., 2004 (24)). As a man of his times, he wrote about women in their roles as housewives, a role he valued highly. Tellingly, he grouped women with ‘minority groups, immigrant workers, lower class persons, and illiterates’, a lack of differentiation of all these groups that would hardly resonate today (14:99).

Antonovsky also wrote as *a man of ideas*, some of which he invited others to test, seemingly having too little time or opportunity to engage in the empirical work himself. In many other instances, he referred to empirical support for his ideas, emanating from his own and others’ research. When it comes to the culture/health relationship, a strong empirical knowledge base has been built over the decades since his passing (25). Two aspects in particular are mentioned here: the experience of cultural integration vs discrimination due to being part of a minority group, and the experience of cultural stability vs instability.

The importance to well-being of being culturally integrated, for instance by taking part in social activities, was recognised by Antonovsky quite early in his career (13). This fundamental insight continues to find empirical support. Four decades later, Forssén (26) observed that elderly women who participated in cultural activities such as theatre and concerts experienced increased strength, comfort and self-esteem, and reduced feelings of loneliness. While taking part in cultural activities may promote integration and the experience of cultural stability, social participation in mainstream cultural life is arduous for many who live on the margins of the mainstream, such as those having a minority background, experiencing cultural discrimination, and having no access to cultural institutions or not having a say in these. Immigrants, as a prime example, may simultaneously face the stress of finding new employment, feelings of loss of social status, loneliness and social isolation, language barriers, culture shock, loss of socioeconomic status, prejudice and isolation (27). Minority groups often have inadequate access to healthcare (28), lower chances of survival following from healthcare deficits (29), and poorer overall health related to poor acculturation and discrimination (30). Directly related to the SMH, Antonovsky wrote that being part of a minority, especially of a minority that is not accepted by the majority, inhibits a strong SOC and thereby threatens health (16). Consistent with this, Braun-Lewensohn and Sagy (31) observed that the Jewish adolescent majority in a study in Israel had a stronger SOC and lower anxiety than Muslims and Druze adolescents, following a traumatic community event (a fire). Further, SOC was stronger among Druze than among Muslims, who are a minority in a majority, and the authors offer the explanation that the Druze are accepted by the Jewish majority and are more integrated in society than the Muslims (31).

On the issue of cultural stability vs instability, Antonovsky viewed cultural stability as the most crucial GRR (2–4,6,32). He maintained that cultural stability leads to a strong SOC, whereas cultural instability and rapid culture change lead to a weak SOC (19,32). Consistent with this, Braun-Lewensohn and Sagy’s (33) study of adolescents in Israel observed that living in a stable community within a multicultural environment protected youngsters from stressors and lead to comprehensibility. The same study found that the SOC of Jewish adolescents living in a stable culture was stronger than the SOC of Arab Bedouins in a nomadic society with instability and inconsistency.

## Conclusion

The paragraphs above link some of Antonovsky’s main ideas about culture and salutogenesis to a few pieces of selected research, keeping within the space constraints of this paper. Obviously, it is well beyond the present scope to evaluate the extent to which theoretical and empirical developments in the era after Antonovsky’s death support or detract from his understanding of culture in the context of salutogenesis. Our much more modest aim was to provide a heretofore unavailable synopsis of what Antonovsky wrote about the roles of culture in salutogenesis. The motivation for this arose from our realisation that culture and its influence on the SOC – and thereby on well-being – was a key, but disjointed theme in Antonovsky’s writings. Our main conclusion is that Antonovsky understood culture as framing life situations which give rise to stressors and to resources, to roles, to meaning, to manageability and to perceptions of health. Of fundamental importance to understanding the SMH, therefore, is an appreciation of cultural factors both as sources of *strength* – GRRs – and as sources both acute and life-course *stress*.

## Notes

i. The database is freely available at the Department of Health Promotion and Development, University of Bergen, at http://www.uib.no/hemil/en; see contents at the Resources tab.

ii. By ‘messages’, Antonovsky meant signals emanating from the social environment, experienced by those exposed to a message as information with meaningful content, or merely uninterpretable noise (14).

iii. Box F in Figure 3 contains the concept well-being, acknowledging the meta-concept well-being within which Antonovsky positioned the health ease/dis-ease continuum.
